# The Level of Selenium and Oxidative Stress in Workers Chronically Exposed to Lead

**DOI:** 10.1007/s12011-015-0435-z

**Published:** 2015-07-17

**Authors:** Natalia Pawlas, Michał Dobrakowski, Aleksandra Kasperczyk, Agnieszka Kozłowska, Agnieszka Mikołajczyk, Sławomir Kasperczyk

**Affiliations:** Institute of Occupational Medicine and Environmental Health, Poland, 41-200 Sosnowiec, Poland; Department of Biochemistry, School of Medicine with the Division of Dentistry in Zabrze, Medical University of Silesia, Katowice, Jordana 19, 41-808 Zabrze, Poland

**Keywords:** Lead poisoning, Selenium, Oxidative stress, Antioxidant defense system, Glutathione peroxidase

## Abstract

The possible beneficial role of selenium (Se) on the oxidative stress induced by lead (Pb) is still unclear in humans. Therefore, the aim of the present study was to explore the associations among the Se levels, chronic Pb exposure, oxidative stress parameters, and parameters characterizing the function of the antioxidant defense system in men who are occupationally exposed to Pb. Based on the median serum Se concentrations, the 324 study subjects were divided into two subgroups: a subgroup with a low Se level (L-Se) and a subgroup with a high Se level (H-Se). The levels of lead (PbB) and zinc protoporphyrin (ZPP) in the blood and the delta-aminolevulinic acid (ALA) level in the urine served as indices of Pb exposure. The PbB level was significantly lower in the H-Se group compared to that in the L-Se group by 6 %. The levels of 8-hydroxyguanosine and lipofuscin (LPS) and the activity of superoxide dismutase were significantly lower in the H-Se group compared to that in the L-Se group by 17, 19, and 11 %, respectively. However, the glutathione level (GSH) and the activities of glutathione peroxidase (GPx) and catalase were significantly higher by 9, 23, and 3 %. Spearman correlations showed positive associations between the Se level and GPx activity and GSH level. A lower serum Se level in chronically Pb-exposed subjects is associated with higher Pb blood levels and an elevated erythrocyte LPS level, which reflects the intensity of oxidative stress. Besides, in a group of Pb-exposed subjects with lower serum Se level, depleted GSH pool and decreased activity of GPx in erythrocytes were reported. However, the present results are inadequate to recommend Se supplementation for chronic lead exposure at higher doses than would be included in a normal diet except for selenium deficiency.

## Introduction

Exposure to lead (Pb) is still a major concern for humans worldwide. Exposure to Pb appears from contact with Pb-based paints, automobiles, batteries, and other sources [1, 2]. The respiratory and gastrointestinal tracts are the principal routes that permit Pb to enter the human body. Pb accumulates in the blood, soft tissues, and bones [3]. According to the newest researches, no safe level of exposure to Pb has been found [4]. The symptoms of the early stages of inorganic Pb exposure include a loss of appetite, weight loss, fatigue, irritability, occasional vomiting, and anemia, whereas at high levels, exposure to Pb causes kidney damage, behavioral disturbances, anemia, hypertension, and toxicity to the reproductive system [1, 2].

The toxicity of Pb is largely due to its ability to induce an imbalance in the generation and removal of reactive oxygen species (ROS) in tissue and cellular components, generally called oxidative stress [5]. On the one hand, the presence of Pb may increase the generation of ROS [6, 7]. On the other hand, Pb depletes the antioxidant defenses, such as glutathione (GSH) and modifies activities and expression of antioxidant enzymes, such as superoxide dismutase or glutathione peroxidase (GPx) [8, 9]. Therefore, exposure to Pb results in the oxidative damage to lipids, DNA, and proteins [5]. The toxic action of Pb is also related to its inhibitory effect on delta-aminolevulinic acid dehydratase (ALAD) and ferrochelatase. Consequently, Pb exposure impairs the chain reaction that leads to the formation of heme and results in the accumulation of delta-aminolevulinic acid (ALA), which has pro-oxidative and alkylating properties [5, 6].

It has been well established that Pb interacts and competes with other cations [10]. Among them is selenium (Se). Se is a non-metal element that has been linked to many health benefits in humans and other mammals such as decreasing the incidence of cancer, protecting against cardiovascular diseases, and treating certain muscle disorders. Besides, Se is involved in mammalian development, boosting immune function, tissue respiration, and antioxidant defense [1, 11, 12]. Se is a cofactor for GPx, an antioxidant enzyme, which utilizes hydrogen peroxide to convert reduced glutathione to its oxidized form (GSSG). It has been demonstrated that selenoproteins, through their antioxidant properties, help to eliminate reactive oxygen species induced by metals [1]. The results of several animal studies have shown that Se or the combination of Se and other antioxidants can reduce Pb-induced oxidative stress. On the other hand, the possible beneficial role of Se alone on Pb-induced oxidative stress is still unclear in humans [5].

The current therapeutic approach to Pb poisoning is to increase its excretion by chelation. Chelators have been shown to reduce blood Pb levels; however, their safety and efficacy may be questioned. Consequently, it is necessary to find new agents capable of abating toxic effects of Pb [13]. In light of this, Se supplementation may be considered as a potential therapy for chronic Pb poisoning due to the antioxidant properties of Se. However, too little is known about possible interactions between Pb and Se. Therefore, the aim of the present study was to explore the associations among the Se levels, chronic Pb exposure, oxidative stress parameters, and parameters characterizing the function of the antioxidant defense system in men who were occupationally exposed to Pb.

## Materials and Methods

### Study Population

The exposed population consisted of 324 occupationally Pb-exposed males who had given informed consent for their participation in this study. All of the study subjects were employed in the zinc and Pb works in the southern region of Poland. They lived near the workplace and had a similar socioeconomic status. The mean concentration of Pb in the air at participants’ workplaces was 0.083 ± 0.12 mg/m^3^. This mean concentration exceeds the Polish MAC level of 0.050 mg/m^3^ [14]. Besides, examined subjects were exposed to negligible doses of zinc. The mean concentration of zinc in the air at participants’ workplaces was 0.15 ± 0.13 mg/m^3^. This value is much lower than the Polish MAC level of 5.0 mg/m^3^. Examined subjects were not occupationally exposed to selenium. All participants continued to work during the study.

Based on the median serum Se concentrations, the subjects were divided into two subgroups: a subgroup with a low Se level (L-Se) and a subgroup with a high Se level (H-Se). Questionnaire data on age, weight, height, medical history, and smoking habits were obtained. Moreover, blood pressure was measured in all of the study subjects. The study was approved by the Bioethics Committee of the Institute of Occupational Medicine and Environmental Health, Sosnowiec, Poland.

The levels of Pb (PbB) and zinc protoporphyrin (ZPP) in the blood and the ALA level in the urine served as short-term lead-exposure indices. To determine the long-term Pb exposure, the concentrations of Pb and ZPP in the blood samples were determined, on average, every 3 months during the 5 years of observation. From the collected data, the mean blood concentrations of Pb (PbB_mean_) and ZPP (ZPP_mean_) were calculated.

### Sampling and Laboratory Procedures

Two samples of blood were obtained from the cubital vein into vacuum tubes (Vacuette®, Greiner-Bio, Frickhausen, Germany) containing K3EDTA for whole blood analyses and plain tubes for serum analyses. Blood samples were frozen and stored at −20 °C until the analyses were performed. Midstream morning urine was collected to measure ALA levels.

To measure the ALA and 8-hydroxydeoxyguanosine (8-OHdG) levels, urine was collected on the same day as blood. The assessments of the PbB and serum Se concentrations were performed by graphite furnace atomic absorption spectrometry [15, 16] using an ICE 3400 instrument (Thermo Fisher Scientific Waltham, MA, USA). The laboratory met the requirements of proficiency tests (Lead and Multielement Proficiency—CDC in Atlanta). The ClinCal® Whole Blood Calibrator and ClinCal® Serum Calibrator (Recipe, Germany) were used for calibration of the instrument and control materials. ClinCheck Whole Blood Control Levels I, II, and III and ClinCheck Serum Control Levels I and II were used for quality control. The results were expressed as micrograms per deciliter (Pb) and micrograms per liter (Se). The level of ZPP was measured using an Aviv Biochemical hematofluorometer, model 206. The results were expressed as micrograms per gram of hemoglobin (Hb). The concentration of Hb was determined using the cyanmethemoglobin method.

The level of ALA was estimated spectrophotometrically in urine samples by the method described by Grabecki et al. [17]. The results were expressed as milligrams per deciliter. The 8-OHdG level was measured in urine using a commercially available ELISA-based assay (Cat. RSCN213100R, BioVendor) according to the manufacturer’s instructions. Spectrophotometric readings were obtained using a BIO-TEK Eon microplate reader (BIO-TEK Instruments), and the results were expressed as grams of creatinine in urine.

The levels of serum malondialdehyde (MDA) were determined according to the method described by Ohkawa et al. [18]. The results were recorded as micromoles per liter. The method developed by Södergren et al. [19] was used to measure the concentrations of lipid hydroperoxides (LPH) in the serum. The results were recorded as micromoles per liter. The levels of lipofuscin (LPS) in erythrocytes were measured according to the Jain [20] method. The results were expressed as relative units (RU) per gram of hemoglobin (the fluorescence of a 0.1 mg/ml solution of quinidine sulfate in sulfuric acid is equal to 100 RU). The total oxidant status (TOS) was measured in serum according to the method described by Erel [21]. The data were shown in micromoles per liter.

The total antioxidant capacity (TAC) in millimoles per liter was measured according to the report by Erel [22]. The levels of erythrocyte glutathione (GSH) were measured as described by Pawelski [23]. The obtained concentrations were expressed as micromoles per gram of Hb (μmol/g Hb). Oyanagui’s method [24] was used to measure the activity of superoxide dismutase (SOD) in erythrocytes. The enzymatic activity of SOD was expressed in nitric units. The activity of SOD is equal to 1 nitric unit (NU) when it inhibits nitric ion production by 50 %. The activities of SOD were normalized to milligrams of Hb (NU/mg Hb). The catalase (CAT) in erythrocytes was measured by the Aebi [25] kinetic method. Catalase activity was expressed as international units per milligram of Hb (IU/mg Hb). The erythrocyte glutathione peroxidase (GPx) activity was measured by the kinetic method described by Paglia and Valentine [26]. The activity of GPx was expressed as micromoles of NADPH oxidized per minute normalized to grams of Hb (IU/g Hb). The activity of glutathione reductase (GR) in erythrocytes was measured according to the report by Richterich [27]. The activity of GR was expressed as micromoles of NADPH utilized per minute normalized to 1 gram of Hb (IU/g Hb).

### Statistical Analysis

The statistical analysis was performed using the Statistica 9.1 PL software program. The statistical analyses included the means and standard deviations of the data. Shapiro-Wilk’s test was used to verify normality, and Levene’s test was used to verify the homogeneity of variances. Statistical comparisons were made using the *t* test, *t* test with separate variance estimates, the Mann-Whitney *U* test, or the chi-squared test. The Spearman non-parametric correlation was calculated. A value of *p* < 0.05 was considered to be significant.

## Results

There were no significant differences in age, height, weight, number of years worked, or smoking habits between the examined subgroups. The percentages of subjects diagnosed with coronary artery disease, hypertension, and diabetes did not differ between the examined subgroups. There was a tendency for the systolic blood pressure to be lower in the H-Se group compared to that in the L-Se group (*p* = 0.058), whereas diastolic blood pressure did not differ between the subgroups (Tables 1 and 2).

**Table 1:** Epidemiologic parameters and the blood lead level (PbB), mean, maximum and minimum PbB, zinc protoporphyrin concentration in blood (ZPP) mean, maximum and minimum ZPP and delta-aminolevulinic acid (ALA) in urine

	Low Se group *n* = 162		High Se group *n* = 162		Relative change (%)	*p* value
	Mean	SD	Mean	SD		
Age	39.0	11.0	38.0	9.78	−2	0.418
Years of work	13.7	11.4	12.0	10.4	−13	0.157
Height (cm)	176	6.32	176	5.83	0	0.608
Weight (kg)	83.8	14.8	85.4	12.4	2	0.308
Systolic blood pressure (mmHg)	121.91	5.52	121.17	5.42	−1	0.058
Diastolic blood pressure (mmHg)	78.52	4.66	78.09	4.93	−1	0.418
PbB (μg/dl)	33.83	9.43	31.85	9.87	−6	0.030
PbB_mean_ (μg/dl)	32.55	8.86	30.87	9.22	−5	0.069
PbB_min_ (μg/dl)	20.12	9.71	19.20	9.42	−5	0.397
PbB_max_ (μg/dl)	43.18	11.47	41.45	12.12	−4	0.087
ZPP (μg/g Hb)	4.28	2.25	4.05	1.90	−5	0.324
ZPP_mean_ (μg/g Hb)	4.81	2.38	4.50	2.26	−6	0.237
ZPP_min_ (μg/g Hb)	2.87	1.52	2.77	1.19	−4	0.512
ZPP_max_ (μg/g Hb)	7.89	4.72	7.36	5.00	−7	0.089
ALA (mg/l)	5.38	1.73	4.77	1.95	−11	0.386

*PbB* and *ZPP* mean levels of Pb and ZPP in the blood determined simultaneously with the biochemical analysis; *PbB*_*mean*_ and *ZPP*_*mean*_ mean levels of Pb and ZPP in the blood calculated based on the values determined, on average, every 3 months during the 5 years of observation before the study; *PbB*_*min*_, *PbB*_*max*_, *ZPP*_*min*_, and *ZPP*_*max*_ mean of the maximal and minimal levels of Pb and ZPP in the blood collected from each worker calculated based on the values determined, on average, every 3 months during the 5 years of observation

**Table 2:** Epidemiologic parameters in study population

	Low Se group (%)	High Se group (%)	*p* value
Smokers	42	34	0.138
Coronary heart disease	2	3	0.476
Hypertension	9	10	0.711
Diabetes	2	4	0.522

**Fig. 1:**
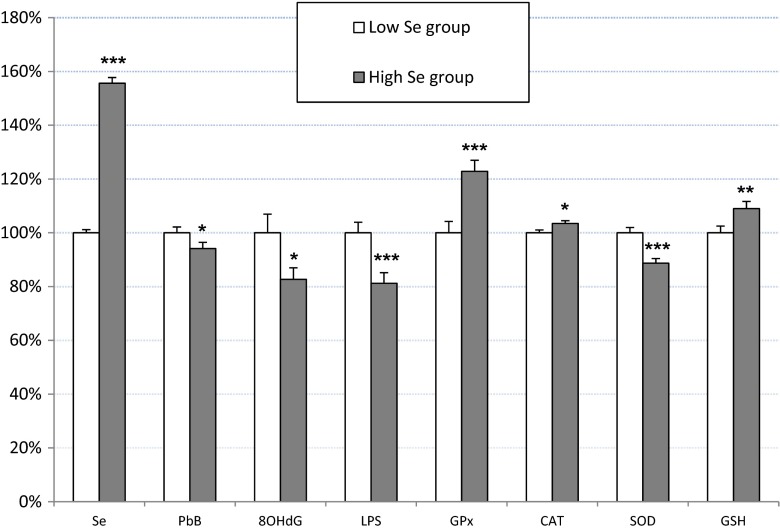
The concentration of selenium (Se) in serum, blood lead level (PbB), 8-hydroxydeoxyguanosine (8-OHdG) in urine, lipofuscin (LPS) in erythrocytes, the activity of glutathione peroxidase (GPx), catalase (CAT), and superoxide dismutase (SOD) in erythrocytes and erythrocyte glutathione (GSH) concentration expressed as a percentage of the mean baseline values in low Se group. Data are presented as a mean and SEM. *p < 0.05, **p < 0.01, ***p < 0.001—comparison between the low and high Se groups

The PbB level was significantly lower in the H-Se group compared to that in the L-Se group by 6 %. At the same time, there was a tendency for there to be lower maximal values of PbB (PbB_max_) and ZPP (ZPP_max_) and for the PbB_mean_ in the H-Se group compared to that in the L-Se group. The other Pb-exposure indices did not differ significantly between the subgroups, although the mean values were lower in H-Se group (Table 1, Fig. 1).

The levels of 8-OHdG and LPS and the SOD activity were significantly lower in the H-Se group compared to that in the L-Se group by 17, 19, and 11 %, respectively. However, the GSH level and activities of GPx and CAT were significantly higher by 9, 23, and 3 %, respectively (Table 3, Fig. 1).

**Table 3:** Concentration of selenium and parameters related to oxidative stress intensity and antioxidant reserves in blood and urine

	Low Se group *n* = 162		High Se group *n* = 162		Relative change (%)	*p* value
	Mean	SD	Mean	SD		
Se (μg/l) in serum	57.9	8.62	90.1	15.6	56	<0.001
TAC (mmol/l) in serum	0.90	0.14	0.92	0.13	3	0.123
TOS (μmol/l) in serum	13.4	15.1	12.0	6.50	−11	0.268
LPH (μmol/l) in serum	4.47	6.07	4.93	3.13	10	0.400
MDA (μmol/l) in serum	2.48	1.06	2.39	1.09	−3	0.469
8OHdG (ng/g creatinine) in urine	13.3	11.73	11.0	7.23	−17	0.039
LPS (RF/dl) in erythrocytes	19.2	9.60	15.6	9.49	−19	<0.001
GPx (U/gHb) in erythrocytes	51.2	27.37	62.9	26.8	23	<0.001
GR (U/g Hb) in erythrocytes	4.59	1.94	4.51	1.63	−2	0.671
CAT (kU/g Hb) in erythrocytes	435	59.0	450	57.4	3	0.023
SOD (NU/mg Hb) in erythrocytes	10.6	2.68	9.40	2.37	−11	<0.001
GSH (μmol/g Hb) in erythrocytes	3.44	1.09	3.75	1.16	9	0.006

Spearman correlations showed positive correlations between the Se level and GPx activity (*R* = 0.32, *p* < 0.001), CAT activity (*R* = 0.12, *p* = 0.025), GSH level (*R* = 0.12, *p* = 0.035), and LPH level (*R* = 0.14, *p* = 0.013). However, negative correlations were reported between the Se level and PbB (*R* = −0.13, *p* = 0.019), PbB_mean_ (*R* = −0.12, *p* = 0.026), PbBmax (*R* = −0.14, *p* = 0.013), LPS level (*R* = −0.24, *p* < 0.001), and SOD activity (*R* = −0.24, *p* < 0.001) (Table 4).

**Table 4:** Correlations between selenium and the blood lead level (PbB), mean, maximum, minimum PbB, zinc protoporphyrin concentration in blood (ZPP) mean, maximum, minimum ZPP, and parameters related to oxidative stress intensity and antioxidant reserves in blood and urine (Spearman R coefficient)

	*R*	*p* value
Se and PbB	**−**0.13	0.019
Se and PbB_mean_	**−**0.12	0.026
Se and PbB_min_	−0.09	0.113
Se and PbB_max_	**−**0.14	0.013
Se and ZPP	−0.02	0.768
Se and ZPP_mean_	−0.08	0.172
Se and ZPP_max_	−0.09	0.110
Se and ZPP_min_	−0.03	0.573
Se and TAC	0.07	0.185
Se and TOS	−0.02	0.721
Se and LPH	0.14	0.013
Se and MDA	−0.07	0.197
Se and LPS	**−**0.24	**<**0.001
Se and 8OHdG	−0.09	0.111
Se and GPx	0.32	**<**0.001
Se and GR	0.04	0.447
Se and CAT	0.12	0.025
Se and SOD	**−**0.24	**<**0.001
Se and GSH	0.12	0.035

## Discussion

The Se concentration in human serum can be divided into three categories: low Se level below 50–60 μg/l, intermediate level between 60 and 100 μg/l, and high level above 100–120 μg/l [28]. In view of this classification, the Se level in the L-Se group can be classified as rather low (57.9 ± 8.62 μg/l) and in the H-Se group (90.1 ± 15.6 μg/l) as intermediate.

The results of the present study indicate that lower Se levels are associated with slightly higher levels of Pb in the blood. The value of PbB, a short-term marker of exposure, was most significantly related to the Se level. An inverse relationship between the levels of Pb and Se was also reported in other studies. For example, in a study by Kasperczyk et al. [10], a lower level of Se was reported in Pb-exposed workers compared with that in unexposed controls. Similarly, Gustafson et al. [29] showed significantly lower plasma Se levels in Pb smelters than in the control group. There was also a significant negative correlation between the PbB and plasma Se level in this study. Additionally, negative correlations between the levels of Pb and Se were shown in children from urban areas in Poland [30, 31] and Senegal [32]. The above-mentioned associations between Pb and Se may be a result of the formation of inactive Pb-Se complexes (Pb selenide) and alterations of the absorption and tissue distribution of Pb by Se [30, 33].

The antagonistic relationship between Pb and Se has been confirmed in experimental studies on animals. Optimal doses of dietary Se were able to partially ameliorate the toxic effects of Pb in poisoned rats, as estimated by measuring the urine ALA level and ALAD activity and Pb level in selected tissues [34]. Moreover, Se was able to mitigate the toxic effects of Pb on the rat brain [33, 35] and to protect the rat submandibular gland function from Pb-induced adverse effects, which were measured by the levels of calcium (Ca) and protein and the activity of *N*-acetyl-β-d-glucosaminidase (NAG) in submandibular secretions [35].

The protective effect of Se in Pb poisoning may be due not only to the above-mentioned mechanisms but also to the reduction of oxidative stress through the antioxidant properties of Se. The decreased level of erythrocyte LPS and increased level of erythrocyte GSH in the H-Se group compared to that in the L-Se group support this hypothesis. Moreover, the Se level was negatively correlated with the LPS level and positively correlated with the GSH level. It is well documented that Pb exposure is associated with GSH depletion as a result of elevated oxidative stress, as well as the accumulation of LPS, which is composed of cross-linked aggregates of oxidized proteins and lipids [8, 36]. Therefore, the higher level of GSH and lower level of LPS observed in the H-Se subgroup can be interpreted as effects of the reduced oxidative stress and may be due to the simultaneously elevated activity of GPx in this subgroup. We also observed a significant positive correlation between the Se level and GPx activity, which suggests that Se deficiency may significantly diminish the antioxidant defense. Consistent with our present findings, in the above-mentioned study on Senegalese children [32], a positive correlation was also shown between the Se levels and GPx activity (*R* = 0.308, *p* < 0.01).

Nevertheless, we did not observe any positive effects of higher Se levels on oxidative stress markers measured in the serum. We showed that there was only a weak positive correlation between the Se level and serum LPH level (*R* = 0.14). Moreover, we did not find any significant differences between the serum values of TAC and TOS and the serum levels of MDA and LPH between the examined subgroups. These results suggest that the bioavailability of Se and its protective effects may be higher in erythrocytes than in serum. This hypothesis is supported by a previous in vitro study showing that the presence of Pb results in the accumulation of Se in erythrocytes [37]. The authors of that study postulated the existence of a protein that simultaneously binds Pb and Se in erythrocytes. Supporting that previous study, Chiba et al. [38] showed that the plasma Se level had a tendency to decrease, whereas its concentration in erythrocytes increased significantly, with an increasing blood Pb level in Pb-exposed workers.

The interactions among the Pb and Se levels and activities of SOD, CAT, and GR are more difficult to interpret. The accumulated data suggest that Pb may trigger opposing mechanisms influencing antioxidant enzyme activities. These complex interactions likely resulted in the divergent results of studies on this topic [9]. In a study by Kasperczyk et al. [9] conducted on Pb-exposed workers, it was shown that Pb exposure induced the expression and activity of SOD as a result of a compensatory defense mechanism against oxidative stress. In light of this finding, the lower level of SOD in the H-Se subgroup compared to that in the L-Se subgroup may be due to a lower intensity of oxidative stress due to the simultaneously elevated activity of GPx and CAT.

The results of the present study are generally supported by other studies. Li et al. [39] showed that Se has protective activities against Pb-induced neurotoxicity in *Caenorhabditis elegans* through its ability to decrease ROS production. Sodium selenite was reported to alleviate oxidative stress injury, as measured by the MDA level induced by Pb exposure, in rats [40]. In other study on rats, Se administration decreased lipid peroxidation in the liver and kidney cells by increasing the activities of the superoxide dismutase and glutathione reductase and the glutathione content [41]. The results of studies on Pb-poisoned fish also confirmed the beneficial effects of Se administration of Pb toxicity [1, 42, 43].

The lower level of urine 8-OHdG in the H-Se subgroup compared to that in the L-Se subgroup in the present study may also be associated with reduced oxidative stress in subjects with higher Se levels. Lead exposure has been linked to elevated DNA damage, including oxidative damage [44]. 8-OHdG is one of the markers of oxidative DNA damage [45], and its lower levels in the H-Se subgroup may be a result of elevated activity of GPx. However, many other mechanisms may be involved, including the influence of Se on DNA repair [46]. Consistent with this possibility, an experimental study on mice reported that the negative effects of Pb on the DNA structure measured in a comet assay were alleviated by the administration of Se [47].

Many studies have shown that Pb can significantly increase arterial blood pressure. This increase may be caused by the influence of ROS on the metabolism of the endothelium and other cells of the circulatory system under Pb-induced oxidative stress conditions [48, 49]. This hypothesis is supported by a study on Pb-exposed workers, which showed that the arterial blood pressure (both systolic and diastolic) was positively correlated with the MDA and Pb levels and negatively correlated with the GPx activity [49]. These results are in agreement with the present findings, because the L-Se subgroup had a tendency to have higher systolic blood pressure compared to the H-Se subgroup. However, data on the associations between the Se level and blood pressure are still inconclusive, and larger-scale studies are needed to clarify these associations [50].

## Conclusions

A lower serum Se level in chronically Pb-exposed subjects is associated with higher Pb blood levels and an elevated erythrocyte LPS level, which reflects the intensity of oxidative stress. Besides, in a group of Pb-exposed subjects with lower serum Se level, depleted GSH pool and decreased activity of GPx in erythrocytes were reported. However, the present results are inadequate to recommend Se supplementation for chronic lead exposure at higher doses than would be included in a normal diet except for selenium deficiency.
